# Biodegradable Polymeric Foams Based on Modified Castor Oil, Styrene, and Isobornyl Methacrylate

**DOI:** 10.3390/polym13111872

**Published:** 2021-06-04

**Authors:** James Anthony Dicks, Chris Woolard

**Affiliations:** Centre for Materials Engineering, Department of Mechanical Engineering, University of Cape Town, Cape Town 7701, South Africa; chris.woolard@uct.ac.za

**Keywords:** vegetable oil, polymeric foam, castor oil, reactive diluent, isobornyl methacrylate, renewable, biodegradable

## Abstract

The environmental issues of petroleum-derived polymeric foams have necessitated seeking renewable alternatives. This work aims to prepare renewable free-radically polymerized polymeric foams with the ability to biodegrade. Furthermore, this work attempted to incorporate a bio-based reactive diluent, which has not been reported in the literature. The synthesis of maleated castor oil glycerides was performed with products analyzed by Fourier transform infrared spectrometry using attenuated total reflection (ATR-FTIR) and ^1^H nuclear magnetic resonance (^1^H NMR) spectroscopy. Polymeric foams were prepared using maleated castor oil glycerides via free radical copolymerization with styrene and isobornyl methacrylate as reactive diluents. Scanning electron microscopy (SEM) was used to determine anisotropic macrocellular morphology, with log-normal cell diameter distributions. The compressive mechanical and energy absorption properties were investigated; the polymeric foams displayed Young’s modulus up to 26.85 ± 1.07 MPa and strength up to 1.11 ± 0.021 MPa using styrene as the reactive diluent, and Young’s modulus up to 1.38 ± 0.055 MPa and strength up to 0.088 MPa when incorporating isobornyl methacrylate. Furthermore, a thorough analysis of the cellular structure–property relationships was performed, indicating relationships to cell diameter, cell wall thickness and apparent density. The polymeric foams displayed rapid mass loss in an aerobic soil environment with multiple erosion sites revealed by SEM. In conclusion, renewable polymeric foams with excellent compressive properties were achieved using styrene as reactive diluent, but the incorporation of isobornyl methacrylate decreased strength-related properties.

## 1. Introduction

In response to environmental concerns surrounding petroleum-derived polymeric materials several renewable thermosetting alternatives have been developed. A primary feedstock of these alternatives is vegetable oils (VOs) [1,2,3,4,5,6,7,8,9]. The functionalization of VOs to prepare thermosetting polymers through free radical polymerization has been developed for several years. A range of both VOs (e.g., soybean, linseed, tung, and castor oils) and functionalization strategies (e.g., epoxidation, acrylation, methacrylation, and maleation) were employed. However, the use of these modified VOs to prepare polymeric foams has remained relatively limited. Of the polymeric foams prepared using modified VOs, acrylated epoxidized soybean oil (AESO) has received the most attention [10,11,12,13,14,15,16,17]. In the case of AESO-based polymeric foams, either homopolymerization or copolymerization with styrene as a reactive diluent was performed. The use of small, rigid compounds as reactive diluents, e.g., styrene, can be useful to provide hard segments within the polymer matrix, thus increasing its mechanical properties. On the other hand, the use of styrene decreases the bio-based carbon content of the product, which is a volatile organic compound, hazardous air pollutant, and a potential human carcinogen [18,19]. While various bio-based reactive diluents have been developed and employed in VO-derived polymeric materials, to the best of the authors’ knowledge these compounds have yet to be introduced into VO-derived free radically polymerized polymeric foams [20,21]. Isobornyl methacrylate (IBOMA) was identified as a promising candidate as a bio-based reactive diluent due to its fused bicyclic ring that could contribute hard segments to the polymer matrix, and due to its low cost, high bio-based carbon content, and commercial availability.

Efforts by Wang and coworkers [22,23] demonstrated the use of maleated castor oil (MACO) to prepare polymeric foams with styrene as a reactive diluent. Castor oil triglycerides provide a reliable feedstock of high purity fatty acid content (~90% ricinoleic acid moieties), which contain secondary hydroxyl functionality at the C_9_ position. The hydroxyl functionality provided a convenient platform for facile, solvent-free, high-yield acid anhydride esterification with maleic anhydride to produce suitable sites for free radical copolymerization with styrene. Furthermore, the carboxylic acid groups present on the maleate half esters provided convenient functionality for acid-metal carbonate reaction with sodium hydrogen carbonate (NaHCO_3_) to induce foaming through CO_2_ liberation as a byproduct. Several studies have demonstrated the improved mechanical properties of polymers prepared with maleated castor oil glycerides (MACOG), a mixture of maleated mono- and diglycerides, compared to that of MACO [18,24,25,26,27,28]. Indeed, it could be expected that reducing the content of long, flexible fatty acid chains per molecule should improve the strength-related properties and T_g_. This work therefore aims to assess whether the use of MACOG could be a suitable modified VO for the preparation of semi-rigid polymeric foams. This work also aimed to incorporate isobornyl methacrylate (IBOMA) as a bio-based reactive diluent and to assess whether it was a suitable alternative to petroleum-derived reactive diluents such as styrene. Furthermore, an in-depth analysis of the structure–property relationships and energy absorption properties was sought to gain a deeper understanding of the cellular mechanics and provide a basis for product design suitability, which is often lacking in the literature for bio-based polymeric foams.

## 2. Materials and Methods

### 2.1. Materials

Castor oil (B.P. grade) was purchased from a local pharmacy (Dischem, Cape Town, South Africa). Isobornyl methacrylate (IBOMA, technical grade, stabilized with 150 ppm monomethyl ether hydroquinone), styrene (≥99%, stabilized with 4-tert-butylcatechol), glycerol (98%), Ca(OH)_2_ (≥95%), maleic anhydride (MA, 99%), sodium hydrogen carbonate (NaHCO_3_, 99.7%), sodium hydroxide (99.5%), hydroquinone (98%), *N,N*-dimethylaniline (*N*,*N*-DMA, ≥99%), *N,N*-dimethylbenzylamine (*N*,*N*-DMBA, ≥99%), dibenzoyl peroxide (BPO, Luperox A75), surfactant (Ewopal 80), diethyl ether (99%), toluene (99.5%) were purchased from Sigma Aldrich (Modderfontein, South Africa). Oxalic acid, sodium hydroxide, ethanol (99.6%), and anhydrous MgSO_4_ were supplied by Kimix Chemicals, Cape Town, South Africa. Styrene contained 4-tert-butylcatechol stabilizer, which was removed by washing with 2M NaOH twice, followed by sequential washing with distilled water and a 4 wt% NaCl aqueous solution. The product was then dried over anhydrous MgSO_4_ and filtered. All other materials were used as received.

### 2.2. Synthesis of Castor Oil Glycerides and Maleated Castor Oil Glycerides

**Figure 1:**
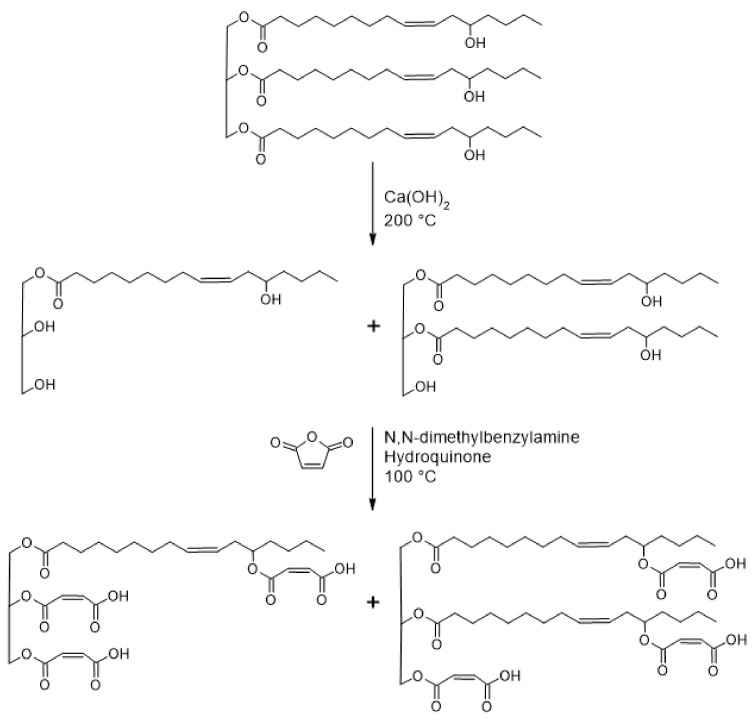
Schematic presentation for the synthesis of MACOG.

The synthesis of castor oil glycerides and maleation of castor oil glycerides was performed by a two-step, “one-pot” synthesis (Figure 1), which was similar to that reported elsewhere [18,24,28]. Details of the syntheses and product characterization can be found in the Supplementary Materials.

### 2.3. Preparation of Polymeric Foams

For PF1 samples, styrene (30 wt% St) was added to MACOG at 65 °C to make up a total mixture of 50.0 g and thoroughly mixed with mechanical stirring under Ar atmosphere. Surfactant (1.5 phr Ewopal 80), accelerant (0.3 phr), and initiator (3 phr BPO) were added to a 400 mL cylindrical reaction vessel. For PF2 samples, the same procedure as PF1 samples was followed, except styrene (15 wt%) and IBOMA (15 wt%) were implemented. For PF3 samples the same procedure as PF1 samples was followed, except IBOMA (50 wt%) was implemented.

Method 1: *N,N*-DMBA was used as the accelerant. The reaction proceeded at 75 °C with mechanical stirring for approximately 20 min. The temperature was then raised to 95 °C, after which hot water (approximately 85 °C) (4 phr) was added and the lid removed. Once the temperature increased to 95–97 °C, foaming agent (0.1–3.0 phr) was added, and the temperature increased to 100 °C as the foam rose and gelled. Curing was performed at 100 °C for 2 h in a convection oven, followed by a post-curing procedure of 120 °C for 2 h and 140 °C for 1 h.

Method 2a: *N,N*-DMA was used as the accelerant. The reaction proceeded for around 10 min at 65 °C until an increase in temperature was noticed due to the polymerization rate increasing. At this point NaHCO_3_ was added and the lid removed. After about 30 s, water was added, and the foam allowed to rise and gel. Once gelled, the sample was placed in a convection oven (Universal Oven, Optolab Zone, Haryana, India) at 100 °C for 2 h, followed by a post-curing procedure of 120 °C for 2 h and 140 °C for 1 h.

Method 2b: The methodology was the same as method 2a, except the reaction was conducted at 75 °C.

The formulation and foaming method are summarized in Table 1 for each of the polymeric foam products.

### 2.4. Characterization Techniques

Acid value (AV) measurements were performed in accordance with ASTM D974-14e2 [29]. A sample (0.5 g) was dissolved in 50 mL of diethyl ether and ethanol mixture (50:50 *v*/*v*), or toluene and ethanol (50:50 *v*/*v*) and 1 mL of phenolphthalein indicator (1 g in 100 mL ethanol) was added to the mixture. The mixture was then titrated against 0.1 N NaOH, which was standardized against 0.1 N oxalic acid standard solution. A blank titration against the solvent mixture was performed, and this titre was subtracted from the titre containing the sample. The *AV* was then calculated according to Equation (1).

(1) $$AV=\frac{56.1\times \left(A-B\right)\times N}{G}\;$$

where *A* is the volume of NaOH solution consumed, *B* is the volume of NaOH solution consumed in a blank test, *N* is the normality of NaOH solution, and *G* the sample mass.

The saponification value (*SV*) was performed in accordance with ASTM D5558-95 [30]. For castor oil, COG, and MACOG, a sample (3 g) was dissolved in 50.0 mL of 0.75 N ethanolic NaOH solution. The mixture was then heated at 78 ± 2 °C under Ar atmosphere and refluxed for 1 h. Once the mixture had cooled, it was back titrated against standardized 0.5 N HCl solution. A blank measurement was performed by the aforementioned procedure without a sample. The *SV* was then calculated according to Equation (2).

(2) $$SV=\frac{\left(A-B\right)\times M_{KOH}\times N_{HCl}}{m}$$

where *A* is the blank titre, *B* is the titre of saponified sample, *M_KOH_* is the molar mass of KOH, *N_HCl_* is the normality of HCl solution, and *m* is the mass of the sample. For both *AV* and *SV* the Ca(OH)_2_ present in COG and MACOG was accounted for in calculations.

^1^H nuclear magnetic resonance (^1^H NMR) spectroscopy was performed using a Bruker Avance III 400 (400 MHz) spectrometer (Bruker, Bremen, Germany). All samples were prepared by dissolution in CDCl_3_. Analysis was performed at 293 K with eight scans per sample, spectral width of 6009.6 Hz, and a pulse width of 10.4000 µs. The data were processed using the MestReNova software package (v. 14.2.0. Mestrelab Research, Santiago de Compostela, Spain).

A Perkin-Elmer Spectrum 100 Fourier transform infrared spectrometer using attenuated total reflection (ATR-FTIR, Waltham, MA, USA) with a universal diamond probe was used to analyze the monomer products, bulk polymers, and polymeric foams. A wavenumber range of 400–4000 cm^−1^ was collected at a resolution of 1 cm^−1^.

The bulk polymer density (ρ_s_) was measured using a pycnometer. The apparent density (ρ*) of the polymeric foams was measured using Vernier calipers (Mitutoyo, Kawasaki, Japan) with a scale resolution of 0.02 mm and the mass was measured using an electronic analytical balance with measurement to the closest 0.0001 g. The porosity (*e*) of the polymeric foam samples was calculated according to Equation (3).

(3) $$e=1-\left(\frac{\rho *}{\rho_{p}}\right)$$

where ρ* is the foam apparent density, *ρ*_p_ is the polymer matrix density.

The polymeric cell morphology was analyzed using a Tescan Mira3 RISE scanning electron microscope (SEM, Brno, Czech Republic). Samples were prepared into rectangles measuring 15 × 15 × 3 mm^3^ and coated with Au-Pd and attached to the stage using a strip of carbon tape. Imaging was performed in secondary electron mode and an accelerating voltage of 5 kV was used with magnifications ranging between ×25 and 60. Cell diameter (D) was calculated both parallel (D_‖_) and transverse (D˫) to the foam rise. Measurements were calculated from SEM micrographs using ImageJ software package (Fiji, v. 1.52), using a minimum of 100 cells. The anisotropy ratio (R) was calculated by *D*_av‖_/*D*_av_˫ [31]. The average cell wall thickness (*δ_av_*) was estimated using Aleksandrov’s equation [32,33] presented in Equation (4).

(4) $$\delta_{av}\approx D_{av}\left(\frac{1}{\sqrt{e}}-1\right)$$

Polymeric foam compressive testing was performed in accordance with ASTM D1621-16 [34], with slight modifications. Compressive testing was performed on a Zwick Roell 1484 Universal Testing Machine (Ulm, Germany) with a 10 kN load cell between two flat platens, and a video extensometer was used to measure the strain. Samples (20 × 20 × 15 mm^3^) were cut on a bandsaw in both parallel and transverse orientations relative to the foaming rise. Sample dimensions were measured using Vernier calipers with a scale resolution of 0.02 mm. The tests were performed at 2 mm/min to a strain of 60%. Recovery was measured after 5 min without stress after 60% strain. Compressive modulus was measured in the linear portion of the initial slope of the stress–strain graph over 1–3% strain. Compressive strength (σ_str_) was measured at 10% strain. Collapse stress (σ_col_) was measured as the first point on the stress–strain graph where $\frac{d\sigma }{d\varepsilon }=0$, or as a tangent to the plateau. The proportional limit (σ_prop_) was measured as the greatest stress where there was proportionality of stress-to-strain (Hookean behavior), measured at the point where the slope of the graph was still >96% of the modulus. The plateau stress (σ_plat_) was measured at 20% strain. The strain at the onset of densification (ε_OD_) was measured by calculating the absorbed energy efficiency (*W_E_*) according to Equation (5), whereby ε_OD_ was calculated at the peak of the graph according to Equation (6). The energy absorbed until onset of densification (W_OD_) was measured according to Equation (7), using the ε_OD_ value measured from Equation (6). [35,36]. *W_to_*_t_ was calculated using the same equation, but changing the upper limit to 60%. σ_plat_, ε_D_, and *W* were calculated using MathWorks MatLab (V. R2020a) by applying a cumulative trapezoidal numerical integration method.

(5) $$W_{E}=\frac{1}{\sigma \left(\varepsilon \right)}\int_{\varepsilon_{y}}^{\varepsilon }\sigma \left(\varepsilon \right)d\varepsilon$$

(6) $$\frac{d\eta \left(\varepsilon \right)}{d\varepsilon }{|}_{\varepsilon =\epsilon_{OD}}=0$$

(7) $$W_{OD}=\int_{0}^{\varepsilon_{OD}}\sigma \left(\varepsilon \right)d\varepsilon$$

### 2.5. Biodegradability of Polymeric Foams

Initially, the polymeric samples (20 × 10 × 10 mm^3^) were cleaned using compressed air to remove any loose particles present from sectioning. Samples were then placed under various biodegradation conditions: (1) An aerobic laboratory soil burial with soil composition similar to that described in ASTM D5988 [37]; (2) a comparative aerobic soil burial in a “natural” environment buried 50 mm in an established compost-rich garden during an austral summer. The relative mass loss was established by periodically removing the samples (removing as much debris as possible with low pressure compressed air) and measuring the mass loss compared to the initial sample mass according to Equation (8).

(8) $$Mass\;loss=\frac{m_{0}-m_{i}}{m_{0}}\times 100$$

where *m*_0_ is the initial mass and *m_i_* is the mass at a given time.

The laboratory control samples were kept in 5 L buckets at ambient temperature, each containing 1 polymeric foam sample and 1 polyurethane (PU) negative control sample, which were sealed with a lid with a small hole (1-mm diameter) to avoid potential CO_2_ gas build-up. The buckets were opened once per week for 15 min to replenish oxygen content. Additionally, the soil was lightly turned monthly while the samples were removed for measurements.

### 2.6. Statistical Analysis

Cell diameter log-normal distribution plots were performed using XLSTAT (Addinsoft, v. 2020.5, Long Island, NY, USA) in Excel 2010. For biodegradation, a Shapiro test for normal distribution was performed, where all data were normally distributed except for month two laboratory environment. Therefore, an unpaired student’s *t*-test was performed for month 1 data and an unpaired two-samples Wilcoxon test for other data. Statistically significant factors were established for *p*-values at a 95% confidence level (*p* < 0.005). All graphs indicate the mean ± standard error of the mean. *AV* and *SV* measurements were an average of three samples. All mechanical and biodegradation results were an average of five samples.

## 3. Results and Discussion

### 3.1. Synthesis of Castor Oil Glycerides and Maleated Castor Oil Glycerides

The base-catalyzed glycerolysis of castor oil was successfully performed to produce COG. The product was characterized by ^1^H NMR (see Supplementary Materials Figure S1a) and ATR-FTIR (see Supplementary Materials Figure S2), with the characteristic ATR-FTIR peak assignments summarized in Table S1 of the Supplementary Materials. The COG produced was then successfully maleated through esterification of the hydroxyl groups with maleic anhydride to produce MACOG. The product was characterized by ^1^H NMR (see Supplementary Materials Figure S1b) and ATR-FTIR (see Supplementary Materials Figure S2), with the characteristic ATR-FTIR peak assignments summarized in Table S1 of the Supplementary Materials. The MACOG product was used directly in the preparation of polymeric foams, without purification.

### 3.2. Preparation of Polymeric Foams

**Figure 2:**
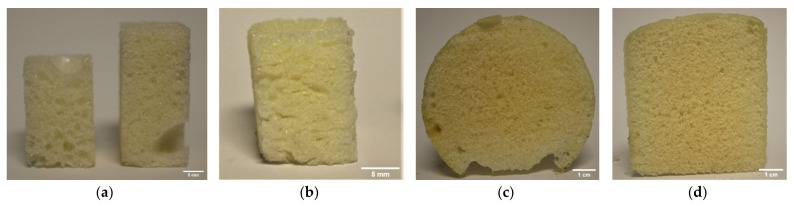
Polymeric foam product with (**a**) coarsening due to early addition of foaming agent, (**b**) cracking due to too late addition of foaming agent, (**c**,**d**) optimized addition of foaming agent.

Thermosetting polymer foams usually need reactive foaming, as the polymer cannot be foamed post-polymerization in the way most thermoplastic polymeric foams are processed [38]. The temperature-temporal relationship between polymer gelling and foaming is therefore critical to ensure optimal foam morphology as well as a high degree of curing. As a result, a delayed addition of foaming agent methodology was implemented. A similar delayed addition of foaming agent has previously been employed by various other groups with improved morphology and properties reported [39,40,41]. The foaming/curing process was not straightforward, and considerable effort was made to optimize and refine the foaming/curing process to achieve polymeric foams with consistent morphology and optimized properties. Two general approaches were taken for the delayed addition of the foaming agent: either to increase the temperature progressively until adding the foaming agent prior to a known temperature of rapid cure was met (method 1), or to maintain a constant temperature for a fixed period until adding the foaming agent when the polymerization reaction increased in rate (method 2). It was found that too early addition of foaming agent could lead to coarsening of the cellular structure as depicted in Figure 2a, or total foam collapse. Conversely, too late addition of foaming agent could lead to cracking as gelling occurred while the foam was still rising, as depicted in Figure 2b. It was thus found that a short window in between these two events could be exploited to achieve an optimized, consistent cell morphology, as depicted in Figure 2c,d. Additionally, it was necessary to employ N,N-DMA as an accelerant when IBOMA was incorporated to maintain practical foam preparation temperatures.

For PF3, several of the methods (methods 1, 2a, 2b) were explored to try successfully preparing polymeric foams. It was found that although stable foams could be formed in the reactive mixture, once cured the polymeric foams lacked any structural integrity and could be readily crumbled. One hypothesis for this effect was that the ester bonds present on IBOMA were highly susceptible to hydrolysis, such that introducing water at elevated temperature to induce foaming caused scission of the monomer. However, waterborne polymerization at 70 °C incorporating IBOMA has been demonstrated without reported hydrolysis, which disputed this hypothesis [42]. It was further speculated that the presence of acid groups on MACOG may have catalyzed the hydrolysis. However, it is also known that methacrylic esters are less susceptible to hydrolysis in both acidic and alkaline environments than acrylic acid esters [43]. In an attempt to minimize the suspected hydrolysis side reaction, a minimum of foaming agent and water were employed (0.15 phr NaHCO_3_ and 0.5 phr H_2_O). However, the polymeric foam still lacked structural integrity and could be easily crumbled. Another hypothesis was that there could have been dissolved oxygen in the water, causing retardation of the polymerization or the formation of low molecular weight polymers. Extensive curing of the polymeric foam at 100 °C for >48 h did not appreciably change the structural integrity of the product though. While the mechanism causing this effect remained unknown, it was concluded that this method of foaming was not suitable to produce PF3.

### 3.3. Polymeric Foam Cell Morphology

**Figure 3:**
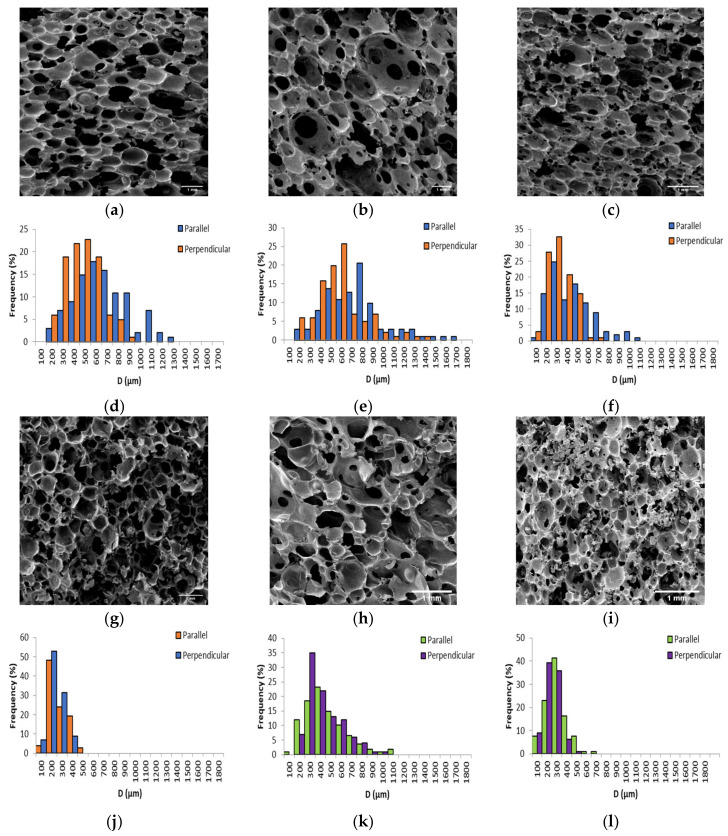
(**a**–**c**) SEM micrograph of PF1a, PF1b, and PF1c, (**d**–**f**) cell diameter distribution of PF1a, PF1b, and PF1c, (**g**–**i**) SEM micrograph of PF1d, PF2a, and PF2b, and (**j**–**l**) cell diameter distribution of PF1d, PF2a, and PF2b.

The apparent density (ρ*), porosity (*e*), average cell diameter (*D_av_*), and average cell wall thickness (*δ_av_*) were measured for PF1 and PF2 samples both parallel and perpendicular to foam rise direction, as summarized in Table 2. SEM micrographs and corresponding cell diameter distributions are presented in Figure 3.

The ρ* of the PFs ranged between 0.126 ± 0.005 g/cm^3^ and 0.309 ± 0.008 g/cm^3^, placing them in the range of medium to high density polymeric foams. This demonstrated that a wide range of ρ* could be tailored by altering the amount of foaming agent employed. Furthermore, the ρ* achieved were in a similar range to many other VO-derived polymeric foams [13,14,17,22,23,44,45]. The *e* ranged between 0.78 and 0.89, which was comparable or higher than that achieved for similar VO-derived polymeric foams [41,44,46,47]. Since it is well-known that the cell morphology of PFs is often anisotropic when allowed to foam freely in one direction, the average cell diameter (*D_av_*) parallel and perpendicular to the foam rise were measured [48].

**Figure 4:**
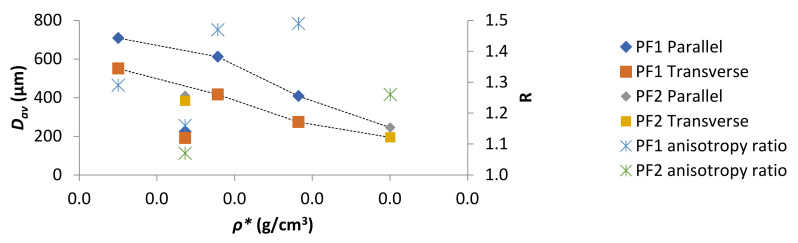
PF *D_av_* and R as a function of ρ*.

The *D_av_* as a function of ρ* was plotted in Figure 4. With the exceptions of PF1d and PF2a (both ρ* 0.168 g/cm^3^) a general decrease in *D_av_* with increasing ρ* was observed for both parallel and perpendicular measurements. However, R values displayed no obvious dependence on ρ*. A particularly good example of such was for PF1d and PF2a, which had the same ρ* but displayed very different R. This difference could have been due to a difference in processing conditions or curing kinetics. However, for PF1 samples, where processing conditions and curing kinetics were kept as similar as possible, there was still a fluctuation in R seemingly independent of ρ*.

**Figure 5:**
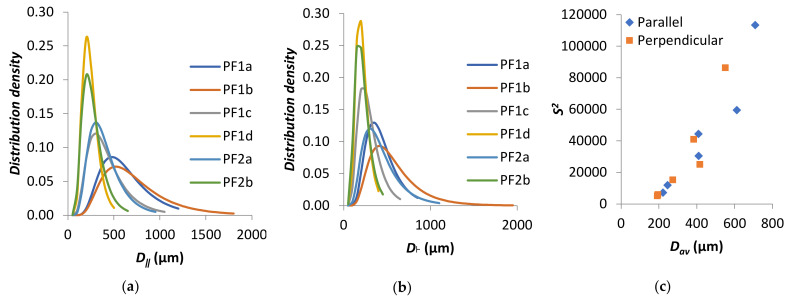
Log-normal density distribution for PF1 and PF2 samples in (**a**) parallel direction and (**b**) perpendicular direction. (**c**) S^2^ as a function of *D_av_*.

The distribution of cell diameters has most commonly been modeled to a log-normal distribution [46,49,50,51,52,53,54]. The results for PF1 and PF2 fit a log-normal distribution, right-skewed toward larger cell diameters, as plotted in Figure 5a,b for the parallel and perpendicular directions, respectively. The variance of the distributions was plotted as a function of *D_av_* in Figure 5c. It was apparent that distributions were generally wider for larger *D_av_*, which was in agreement with other literature [55].

### 3.4. Compressive Properties

**Figure 6:**
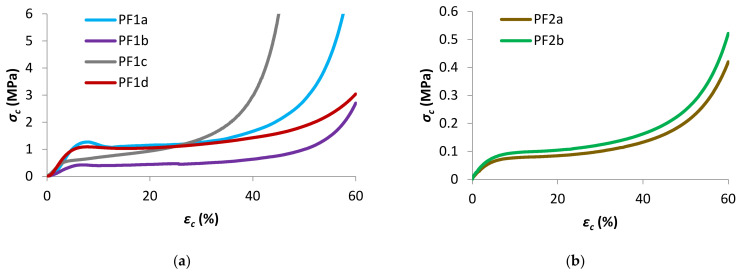
Representative compressive *σ_c_–ε_c_* plots (**a**) PF1 ParFR and (**b**) PF2.

The uniaxial compressive properties of PF1 and PF2 samples were analyzed, with samples compressed parallel to the foam rise (ParFR) direction plotted in Figure 6a,b, respectively. Furthermore, the compressive properties of samples PF1a and PF1b were compressed perpendicular to the foam rise (PerFR). The characteristic compressive properties for the PFs are summarized in Table 3. The compressive properties were measured for both ParFR and PerFR because it was anticipated that the anisotropic cell morphology would result in anisotropic compressive properties [56].

PF1c was prepared using method 2b, as opposed to method 1 that was used for PF1a, b and d. It was apparent that the processing conditions could have affected the overall mechanical properties of the polymeric foams, as in most cases PF1c did not match the relationships that correlated well between PF1a, b, and d. It was for this reason that PF1c was excluded from the relationships in most cases. This phenomenon was considered interesting and therefore the result has been reported, but more PF1 samples would need to be prepared using method 2b to verify that it was the processing conditions that caused the observed discrepancy.

Generally, the compressive properties of the polymeric foams displayed three distinct stages on a *σ_c_–ε_c_* graph: an elastic stage, a plateau stage, and a densification stage. An initial linear elastic region was seen on the *σ_c_–ε_c_* graphs, observed at low strains (*ε_c_* < 5%) for all polymeric foams. This region corresponded to the bending of cell edges and struts, cell membranes stretching (for closed cells) and to a lesser extent gas pressure within closed cells [38,56].

**Figure 7:**
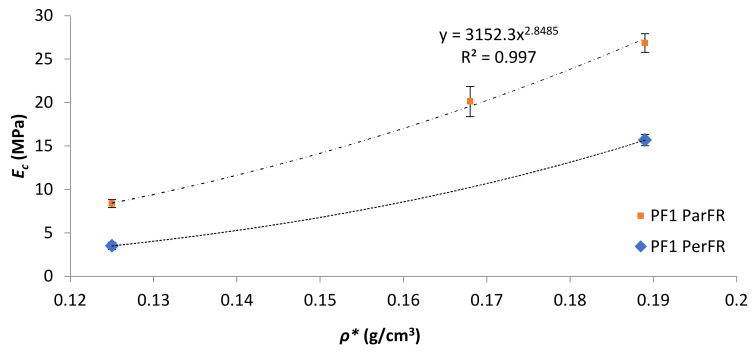
*E_c_* as a function of ρ* for PF1.

The Young’s modulus (*E_c_*) was calculated in this region of the graph, between *ε_c_* of 1–3%. The *E_c_* as a function of ρ* was plotted in Figure 7 for PF1 ParFR and PF1 PerFR. It was evident that *E_c_* increased with increasing ρ* in both ParFR and PerFR; with a maximum *E_c_* of 26.85 ± 1.07 MPa for PF1a ParFR using styrene as reactive diluent, and 1.38 ± 0.055 MPa for PF2b using styrene and IBOMA as reactive diluent. It has previously been established that there is a power–law relationship between *E_c_* and ρ*, according to Equation (9).

*E*_*c*_ = Bρ*^m^ (9)

where ρ* is the apparent density of the PF, *B* is a constant related to the physical properties of the resin, and *m* is a density exponent related to the structure and deformation mechanics of the PF.

PF1 ParFR fit a power–law relationship (R^2^ = 0.9984) with a B value of 3152 MPa and an *m* value of 2.85. Wang et al. [22] calculated a B value of 514 MPa and an *m* value of 3.55 for MACO/St (30 wt% St) polymeric foams. Furthermore, it was reported that both B and *m* values increased with increasing St wt% (10–40 wt% reported) due to the rigid contributions of the stiff, aromatic reactive diluent. Based on the model, the larger B value reported here suggested it was the polymer matrix properties that contributed to the superior results of that of the MACO/St polymeric foams [57]. PF2 E_c_ displayed a far weaker dependence on ρ*, whereby almost doubling ρ* between PF2a (1.13 ± 0.11 MPa) and PF2b (1.38 ± 0.06 MPa) resulted in a marginal increase in *E_c_*. This could have been due to fundamental differences in cell morphology between the samples [58].

**Figure 8:**
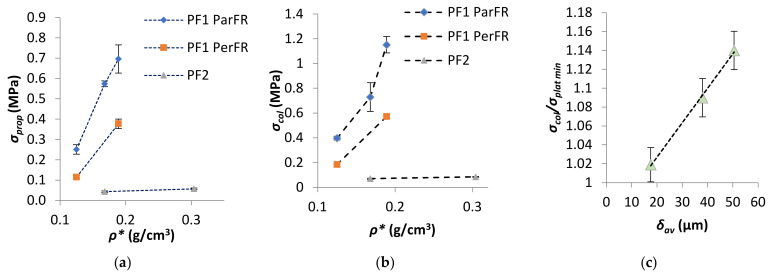
(**a**) *σ_prop_* as a function of ρ* for PF1, (**b**) *σ_col_* as a function of ρ*, and (**c**) ratio of *σ_col_/σ_plat_*_min_ as a function of *δ_av_* for PF1 ParFR.

It was apparent that there was a large difference in *E_c‖_* and *E_c_˫* observed for PF1a and PF1b, as visualized in Figure 7. The difference in *E_c‖_* relative to *E_c_˫* has been reported to be partially influenced by differing deformation mechanisms in anisotropic polymeric foams; whereby parallel to the foam rise there is axial deformation of the cell edges oriented parallel to the loading axis, while perpendicular deformation is carried by axial loading and bending of the cell edges and is hence dependent on the cell edges’ stiffness. This implies that a stiff linear response was seen until cell instability (beyond *σ_prop_*) in parallel, which was not the case for perpendicular, where stress due to cell edges bending increases to a lesser degree with progressive *ε_c_* [59]. Gibson and Ashby [48] developed a model that proposed a direct proportionality between R and *E_‖c_*, such that *E_‖c_* increased with increasing R, since at greater R the walls are relatively longer in the parallel direction. The specific *E_‖c_* of PF1a ParFR (0.142 ± 0.006 MPa·m^3^/kg) was higher than PF1b ParFR (0.067 ± 0.004 MPa·m^3^/kg) and R_PF1a_ > R_Pf1b_, which was in agreement with this model and other literature [31,55,60]. The proportional limit (*σ_prop_*) was measured as the *σ_c_* at which deviation from Hookean behavior relative to *ε_c_* was observed, thus at the onset of plastic compressive behavior. The *σ_prop_* as a function of ρ* was plotted in Figure 8a; and it was evident there was a correlation with increasing *σ_prop_* and increasing ρ*.

The collapse stress (*σ_col_*) was measured at the local peak of the *σ_c_–ε_c_* graph after *σ_prop_* for PF1 ParFR and as a tangent to the plateau for PF1 PerFR and PF2. The *σ_col_* as a function of ρ* was plotted in Figure 8b where it was apparent that there was a correlation between increasing *σ_col_* and increasing ρ*. Furthermore, it appeared that there were two dominant modes of collapse stress: elastic buckling (PF1 PerFR, PF1c ParFR, PF2) and plastic collapse (PF1a, b, d ParFR) [48,61]. This was observed by a region of strain-softening evident for PF1 ParFR (except PF1c), which was not observed for PF1 PerFR and PF2. For PF1 this could have been due to a difference in deformation mechanisms due to cell anisotropy. It has been reported that cell elongation parallel to the compression axis favors plastic collapse, whereby if the fully plastic moment of the cell edge is exceeded, plastic hinging at the strut occurs. On the other hand, cell elongation perpendicular to the compression axis favors elastic buckling, whereby if the Euler load on the cell edge is exceeded buckling occurs, which is proportional to the edges’ flexural stiffness [31]. As a result, when extensive plastic collapse occurred for PF1 ParFR a decrease in the PFs ability to retain load bearing capacity was observed [48,62]. The extent of strain softening has previously been demonstrated to correlate well with ρ* by Lee et al. [63] and Lim et al. [62]. However, this was not evident for PF1 samples; rather, it was found that the extent of strain softening, measured by the ratio of *σ_y_/σ_plat min_*, correlated to the *δ_av_*, as plotted in Figure 8c. Although there is often a relationship between ρ* and *δ_av_*, this is not always the case, as was demonstrated for PF1d and PF2a, which displayed similar density but very different *δ_av_*. During compression, *δ_av‖_* was related to those undergoing axial loading during plastic collapse, while *δ_av_˫* was related to bending of those perpendicular to compression axis, thus *δ_av_* was used in calculation [31,48,59]. PF1c and PF2 did not display strain softening, despite being anisotropic. This could be due to a fundamental difference in polymerization/processing, causing the PFs to respond in a typical elastomeric manner instead of elastoplastically [38]. It was worth noting that while PF2 contained a different polymer matrix to PF1c, both PF2 and PF1c were prepared by method 2a/b as opposed to method 1 for PF1a, b and d.

At *ε_c_* beyond the point of *σ_col_* and strain softening (if apparent), the polymeric foams displayed a plateau stage. During the plateau stage, deformation and stress response were accounted for by cells collapsing via elastic and/or plastic buckling mechanisms, walls and edges being ruptured throughout the material and gas pressure in closed cells. Typical to the plateau stage, low changes in *σ_c_* over a large *ε_c_* range were observed for the polymeric foams, although the plateau moduli were positive in all polymeric foams. One contributing factor to the positive plateau modulus could have been gas pressure within closed cells [38]. In the plateau region deformation can occur through elastomeric, elastoplastic, and brittle modes. For elastomeric deformation, no plastic deformation occurs to cell edges, struts, and walls, and deformation is accounted by a pure elastic buckling mechanism. For elastoplastic deformation there is plastic deformation of the cell edges, struts, and walls. These phenomena are observed differently within the cellular structure; elastomeric response is characterized by uniform distribution of deformation, while elastoplastic is characterized by progressive local deformation of the cell layers acting as “deformation fronts” of the weakest cell layer [56]. It is common for a combination of modes to be present in a polymeric foam. Brittle failure was not observed as the dominant mode during the plateau region for all polymeric foam, which is typically characterized by a jagged σ-ε plot [38]. This suggested that the fatty acid chains on MACOG provided mobility to the polymer.

**Figure 9:**
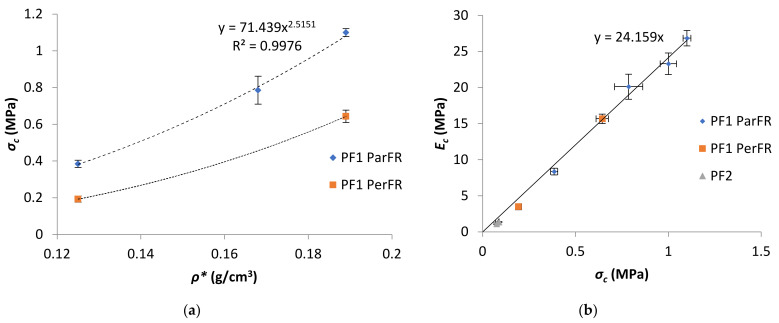
(**a**) *σ_c_* as a function of ρ* for PF1 and (**b**) E_c_ as a function of *σ_c_*.

The *σ_str_*, measured at *ε_c_* of 10% for semi-rigid and rigid polymeric foam, was within the plateau stage for all polymeric foams. It was apparent that *σ_str_* increased with increasing ρ*; with a maximum *σ_str_* of 1.11 ± 0.021 MPa for PF1a ParFR using styrene as the reactive diluent, and 0.088 ± 0.0031 MPa for PF2b using styrene and IBOMA as reactive diluent. There was a power–law relationship between *σ_str_* and ρ* according to Equation (10). This relationship was plotted in Figure 9a for PF1, where a power–law relationship (R^2^ = 0.9985) was fit with an A value of 71 MPa and *n* value of 2.5 for PF1 ParFR.

(10) $$\sigma_{str}=A\rho^{*n}$$

where ρ* is the apparent density of the PF, A is a constant related to the physical properties of the resin, and *n* is a density exponent related to the structure and deformation mechanics of the PF.

According to Gibson and Sanders [64] a theoretical *n* value of 2 relates to closed cell polymeric foams, while *n* values of 1.5 and 1.36 relate to open cell and hollow sphere cell polymeric foams, respectively, and fit well with previous experimental literature [12,57,65,66,67]. However, it was also evident from literature that deviation from the theoretical model with *n* < 2 was not unusual for polymeric foams [22,67]. The *n* value for PF1 ParFR was in agreement with the cellular deviation from spheres associated with porosities greater than 0.63 and majority closed cell content [68].

A linear correlation between *σ_str_* and *E_c_* was established for PF1 ParFR (R^2^ = 0.985), as plotted in Figure 9a, with a slope of 24.2. A linear relationship has also been reported by Bonaillie [12] for AESO polymeric foams with a slope of 17. The larger slope here indicated a greater relative elastic stiffness, which could be partially attributed to the anisotropy of cells and introduction of hard segments from the reactive diluent compounds. Although the compressive moduli and strength of PF2 were substantially lower than those of PF1, these results were still higher than other polymeric foams reported in literature that were produced with a greater weight percentage of styrene. For example, PF2a displayed a *σ_str_* (87.6 ± 3.1 kPa) more than double that of MACO/St (20 wt% St) PF (33.8 ± 1.2 kPa) of similar ρ* produced by Wang et al. [22]. Furthermore, PF2 displayed compressive properties that were comparable to various other bio-based semi-rigid PFs [23,69,70]. However, it was also possible that the issues associated with preparing PF3 could also have contributed to the lower mechanical properties in PF2.

Lastly on the *σ_c_–ε_c_* plot, densification occurred once the cell walls completely collapsed, causing compaction against each other and the material began acting as a homogeneous solid. Densification was denoted by a steep rise in *σ_c_* over a small *ε_c_* [56]. The differentiation between the terms “densification strain” (*ε_D_*) and “onset of densification strain” (*ε_OD_*) has often remained ambiguous or been used interchangeably. However, these two measurements are distinct, whereby the *ε_OD_* occurs when the deformation modes typical of the plateau region are suppressed due to contact between the cell walls and edges, and precedes the *ε_D_*; whereas *ε_D_* is the point where the polymeric foam is completely compacted [35,71]. From a product design application perspective, the *ε_OD_* is a more useful parameter to consider, as it can be used to calculate the energy the polymeric foam is able to absorb before it acts as a homogenous solid [44]. The *ε_OD_* was calculated by implementing the methodology of Tan et al. [36], which was further developed for polymeric cellular solids by Li et al. [35] as a reliable method within literature [40,71,72,73,74,75]. The energy absorption characteristics have been summarized in Table 4.

**Figure 10:**
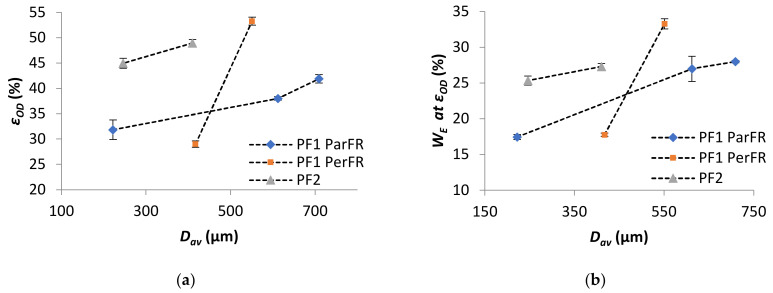
(**a**) *ε_OD_* as a function of *D_av_* and (**b**) *W_E_* as a function of *D_av_*.

Strain at onset of densification (*ε_OD_*) and energy absorption efficiency at onset of densification (*W_E at OD_*) both increased with increasing *D_av_* in all cases, as plotted in Figure 10a,b, respectively. The results of *ε_OD_* increase with *D_av_*, as in agreement with the literature [76]. Furthermore, it was apparent that both the *ε_OD_* and *W_E at OD_* were higher for PF2 than PF1 ParFR.

**Figure 11:**
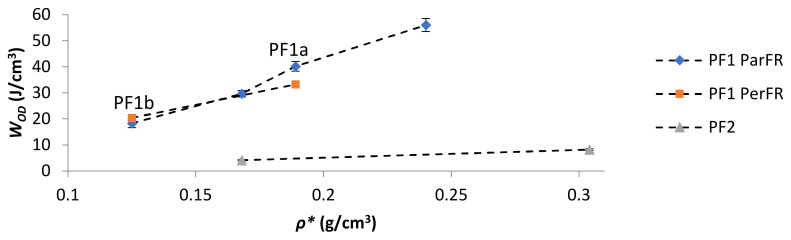
*W_OD_* as a function of ρ*.

The energy absorbed at the onset of densification (*W_OD_*) increased with increasing ρ* in all cases, as plotted in Figure 11. The comparative W_OD_ for PF1b ParFR and PF1b PerFR was similar, while PF1a ParFR was higher than PF1a PerFR. This was particularly interesting, as both the *W_E at OD_* and *ε_OD_* were lower for PF1a PerFR than PF1b PerFR. This result could have arisen due to the differing deformation modes during the plateau region, since PF1a displayed a larger anisotropy than PF1b. Furthermore, the *W_OD_* was higher for PF1 than PF2, even though both *W_E_* and *ε_OD_* were higher for PF2 than PF1. This result suggested that while PF2 was able to absorb energy at both a higher efficiency and until greater strains, the energy absorption was still limited by the properties of the polymer matrix. Furthermore, the deformation modes described during the plateau stage were thought to be similar to that of PF1 PerFR as previously described, which could have further contributed to this result.

**Figure 12:**
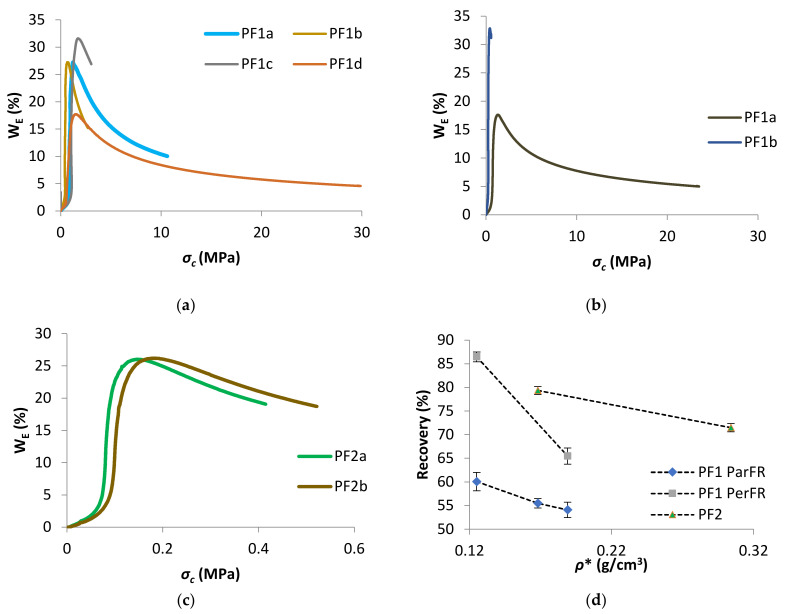
W_E_ as a function of *σ_c_* for (**a**) PF1 ParFR, (**b**) PF1 PerFR, and (**c**) PF2, and (**d**) recovery as a function of ρ* for PF1 ParFR, PF1 PerFR and PF2.

W_E_ as a function of *σ_c_* was plotted in Figure 12a for PF1 ParFR, Figure 12b for PF1 PerFR, and Figure 12c for PF2. There was linear proportionality between *W_E_* and *σ_c_* until the *σ_prop_*. Following the proportional limit, *W_E_* increased rapidly, with a maximum close to *σ_col_*. W_E_ slowly decreased until the *σ_c_* at *ε_OD_*, and then continued to decrease progressively at *σ_c_* beyond *ε_OD_*. A similar relationship between W_E_ and these characteristic *σ* values has been reported in literature [74,75,77,78].

The recovery as a function of ρ* was plotted in Figure 12d, which showed increasing ρ* correlated with decreasing recovery for all polymeric foams. It was possible that the higher recovery displayed by PF1 PerFR was due to dominant elastomeric deformation during the plateau stage, compared to elastoplastic deformation in PF1 ParFR. When stress was removed elastomeric contributions had a greater ability to return to original geometry, while elastoplastic deformation cannot. Likewise, PF2 recovery were higher than PF1 ParFR, which further suggested a dominant elastomeric response to compression [38]. Furthermore, a higher elastic contribution allowing greater recovery could be accounted for a polymer matrix that is more flexible. On the other hand, if the rigid PF1 samples displayed dominant elastoplastic deformation, acting as homogenous solids on densification, they would be less able to recover [46].

### 3.5. Biodegradability

The end-of-life biodegradability of polymeric foams is a highly desirable property, as waste accumulation is mitigated and its reversion into biomass enhances its renewability. While it was noted that measuring mass loss may account for mechanisms of degradation other than microbial biodegradation alone, the use of controlled environments could mitigate the degree of influence of these factors. Furthermore, to minimize any potential damage to the specimen during cleaning for measurements, a small amount of soil was retained on the specimens. A mass loss of 0.34 ± 0.30% was measured for the negative control sample after 2 months in the laboratory environment, which suggested that processing of samples had a negligible contribution.

**Figure 13:**
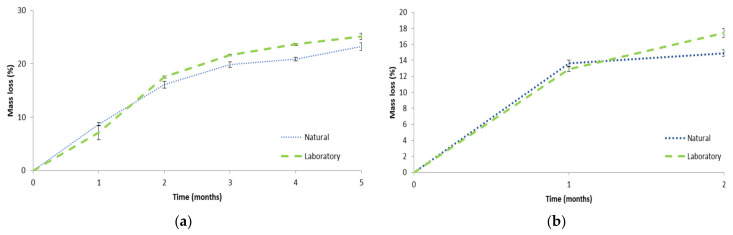
Mass loss of polymeric foam samples in natural and laboratory aerobic biodegradation conditions for (**a**) PF1 and (**b**) PF2.

The mass loss as a function of time is plotted in Figure 13a for PF1 in both laboratory and natural soil burial environments. It was evident that rapid mass loss was achieved, which could be due to the hydrolytically susceptible ester bonds present on MACOG and hydrophilicity of the material promoting microbial colonization [79]. The rate of biodegradation was similar to MACO/styrene (30 wt%) foams as described by Wang et al. [22]. This result was surprising, since it was expected that MACOG/styrene would contain more elastically active chains than MACO/styrene (as previously indicated by Can et al. [25]), which was expected to decrease the rate of biodegradation [80]. Moreover, measurements by Wang et al. [22] were performed at 30 °C, and it is well-known that biodegradation rate increases with temperature [81]. Furthermore, the mass loss was higher than various AESO-based polymeric foams [12,14,17]. This result highlighted the potential of castor oil as a renewable feedstock compared to soybean oil-derived materials. Comparatively, it was interesting that there was no significant difference in mass loss between the two environments for the first three months (month 1: *p* = 0.64, month 2: *p* = 0.55, month 3: *p* = 0.160). Thereafter the laboratory environment was higher in month 4 (*p* = 0.011) but not significantly different after 5 months (*p* = 0.076), reaching 25.1 ± 0.6% and 23.2 ± 0.7% for the laboratory and natural environment, respectively.

The mass loss as a function of time was plotted in Figure 13b for PF2 in both laboratory and natural soil burial environments. The mass loss after 1 month was not significantly different between the two environments (*p* = 0.456), but in month two the laboratory environment was significantly higher than the natural environment (*p* = 0.019). After two months the mass loss for PF2 in the laboratory environment was significantly higher than PF1 month 2 in both the natural (*p* = 0.038) and laboratory (*p* = 0.019) environment. However, PF2 in the natural environment was not significantly different to PF1 month 2 in either the natural (*p* = 0.404) or laboratory (*p* = 0.806) environment. It was anticipated that PF2 may have a greater ability to biodegrade than PF1 owing to the hydrolytically susceptible ester bonds on the isobornyl fragments. However, as this was only observed for PF2 laboratory environment it could not be definitively ascertained whether the addition of IBOMA positively contributed to the biodegradability. Furthermore, it was acknowledged that longer biodegradation time for PF2 would be beneficial to try determining IBOMA’s effect on the biodegradability.

**Figure 14:**
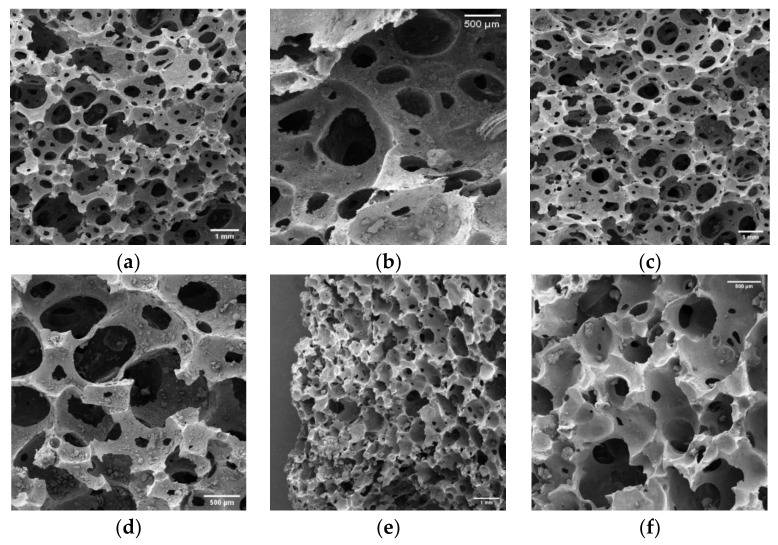
(**a**,**b**) PF1 after 5 months in a natural aerobic soil environment, (**c**,**d**) PF1 after 5 months in a laboratory aerobic soil environment, and (**e**,**f**) PF2 after 2 months in an aerobic soil environment.

SEM micrographs of the cell structure of polymeric foams after biodegradation are presented in Figure 14. Qualitatively, the formation of numerous erosion sites within the internal cellular structure could be observed, while the surface appeared to lose well-defined morphological integrity [22,82,83]. No obvious difference between the laboratory and natural samples could be identified from the images, suggesting that similar mechanisms of degradation occurred in both environments. The presence of small erosion sites within the cellular structure suggested that mass loss due to mechanisms other than mechanical degradation successfully took place, inferring that mass loss was likely due to microbial activity.

## 4. Summary and Conclusions

This work demonstrated that renewable alternatives to petroleum-derived polymeric foams could be accomplished by using modified castor oil and isobornyl methacrylate as feedstocks. The synthesis of MACOG was achieved without the need for solvents or purification, thus providing an efficient and sustainable method of preparing a modified vegetable oil with suitable sites for free-radical polymerization. Furthermore, the carboxylic acid groups on MACOG provided a convenient platform for foaming with NaHCO_3_ as an environmentally benign foaming agent. While the preparation of polymeric foams by reactive foaming was not straightforward, some of the issues were resolved by optimizing the processing conditions to achieve consistent cell morphology. Furthermore, the degree of foaming could be tailored by the amount of NaHCO_3_ employed, hence proving that a range of apparent density and porosity of polymeric foams were achievable with this method.

The resultant polymeric foams displayed anisotropic cell morphology with elongation in the direction of the foam rise and log-normal cell diameter distributions. Cell anisotropy was found to have a strong effect on the compressive properties of the polymeric foams, with improved properties in the direction of cell elongation, which was likely due to differing cellular deformation mechanics in either direction of the compression. This structure–property anisotropy can be exploited in design applications, although control over cell elongation would require further processing optimization. Furthermore, the compressive properties obeyed many other established cellular mechanical models, with structure–property relationships found related to the apparent density and average cell wall thickness. It was found that increasing apparent density was the dominant contributor to increased modulus and strength but decreased the ability to recover. The compressive energy absorption properties indicated that the foams could effectively absorb energy until the onset of densification. More specifically, the cellular structure of the polymeric foams influenced the ability to absorb energy, with greater strains at onset of densification and higher energy absorption efficiency for larger cell diameters. Additionally, the energy absorbed at the onset of densification was observed to increase with increasing apparent density. These relationship parameters provide valuable understanding toward controlling and tailoring the polymeric foams to provide suitable mechanical and energy absorption properties.

Overall, it was evident that both the polymer properties as well as morphological features influenced the compressive deformation mechanisms and hence mechanical and energy absorption properties. It also preemptively appeared that the processing conditions may have had an additional effect on the compressive properties. Comparatively, it was apparent that the choice of reactive diluent strongly influenced the mechanical properties of the polymeric foams. The sole employment of IBOMA as a reactive diluent was not successful in this work. It became apparent that careful consideration of both the relative reactivity of the functional groups and molecular structure of the reactive diluent was needed to provide effective hard segments within the polymer matrix to improve the overall mechanical properties. Indeed, the incorporation of IBOMA appeared to change the compressive cellular mechanics and thus the overall mechanical properties, which were generally lower than polymeric foams only using styrene. However, the energy absorption properties and recovery were comparable or higher than the polymeric foams only employing styrene, with exception to lower energy absorbed at the onset of densification. This work has provided an initial platform for understanding the incorporation of bio-based reactive diluents into VO-derived free-radically polymerized polymeric foams, which until now had not been reported in literature. That being said, the polymeric foams employing only styrene still provided a bio-based product that displayed favorable properties compared to many other bio-based polymeric foams reported in literature. Furthermore, all the polymeric foams displayed the ability to rapidly biodegrade in an aerobic soil environment, which allows for reversion to biomass at the end of lifespan, thus providing an environmentally sustainable material.

## Figures and Tables

**Table 1:** Formulation of polymeric foams.

	Styrene (wt%)	IBOMA (wt%)	NaHCO_3_ (phr)	H_2_O (phr)	Foaming Method
PF1a	30	0	1.75	4	1
PF1b	30	0	3.0	4	1
PF1c	30	0	1.5	4	2a
PF1d	30	0	2.0	4	1
PF2a	15	15	2.25	4	2b
PF2b	15	15	1.5	4	2b
PF3a	0	50	2.25	4	1
PF3b	0	50	0.1	0.5	1, 2a, 2b

**Table 2:** Polymeric foam cell morphology.

	ρ* (g/cm^3^)	*e*	D_av‖_ (µm)	D_av˫_ (µm)	R	*δ_av‖_* (µm)	*δ_av˫_* (µm)
PF1a	0.189 ± 0.003	0.83	612 ± 24	417 ± 16	1.47	60	41
PF1b	0.125 ± 0.006	0.89	709 ± 34	551 ± 29	1.29	43	33
PF1c	0.241 ± 0.007	0.78	409 ± 21	274 ± 12	1.49	54	36
PF1d	0.168 ± 0.004	0.85	222 ± 8	192 ± 7	1.16	19	16
PF2a	0.168 ± 0.004	0.85	410 ± 17	384 ± 19	1.07	35	33
PF2b	0.309 ± 0.008	0.73	246 ± 11	195 ± 7	1.26	42	33

**Table 3:** Compressive properties of PF1 and PF2.

	E_c_ (MPa)	*σ_prop_* (MPa)	*σ_col_* (MPa)	*σ_str_* (MPa)	*σ_plat_* (MPa)	Recovery (%)
PF1a ParFR	26.85 ± 1.07	0.70 ± 0.069	1.15 ± 0.066	1.11 ± 0.021	1.79 ± 0.172	54 ± 1.6
PF1a PerFR	15.69 ± 0.65	0.38 ± 0.023	0.57 ± 0.014	0.64 ± 0.033	0.80 ± 0.033	65 ± 1.7
PF1b ParFR	8.37 ± 0.46	0.25 ± 0.023	0.40 ± 0.015	0.39 ± 0.019	1.25 ± 0.33	60 ± 1.9
PF1b PerFR	3.51 ± 0.43	0.11 ± 0.0079	0.18 ± 0.019	0.19 ± 0.017	0.20 ± 0.017	86 ± 1.0
PF1c	23.31 ± 1.49	0.57 ± 0.011	1.00 ± 0.038	1.01 ± 0.044	1.05 ± 0.064	81 ± 1.0
PF1d	20.13 ± 1.73	0.57 ± 0.14	0.73 ± 0.12	0.79 ± 0.0761	1.01 ± 0.090	56 ± 1.0
PF2a	1.13 ± 0.107	0.048 ± 0.0033	0.072 ± 0.010	0.075 ± 0.0053	0.083 ± 0.0066	79 ± 1.0
PF2b	1.38 ± 0.055	0.057 ± 0.0039	0.085 ± 0.0030	0.088 ± 0.0031	0.092 ± 0.0031	71 ± 0.9

**Table 4:** Energy absorption properties.

	Orientation	*ε_OD_* (%)	*W_E at OD_* (%)	*W_OD_* (J/cm^3^)	*W_tot_* (J/cm^3^)
PF1a	ParFR	38.0 ± 0.364	27.41 ± 0.16	40.22 ± 1.85	124.4 ± 7.93
	PerFR	29.01 ± 0.63	17.77 ± 0.21	20.39 ± 0.96	120.02 ± 1.77
PF1b	ParFR	41.9 ± 0.83	27.35 ± 1.76	18.36 ± 1.68	41.17 ± 5.43
	PerFR	53.28 ± 0.78	33.29 ± 0.71	11.82 ± 0.90	14.61 ± 1.32
PF1c	ParFR	49.16 ± 0.53	30.48 ± 0.63	56.02 ± 2.52	81.53 ± 4.27
PF1d	ParFR	31.86 ± 1.92	17.45 ± 0.34	29.69 ± 1.24	134.66 ± 1.98
PF2a	ParFR	48.97 ± 0.99	27.31 ± 0.43	4.53 ± 0.312	8.22 ± 0.28
PF2b	ParFR	44.69 ± 0.49	25.35 ± 0.64	4.18 ± 0.20	8.18 ± 0.58

## Data Availability

The data presented in this study are available on request from the corresponding author.

## Supplementary Materials

The following are available online at https://www.mdpi.com/article/10.3390/polym13111872/s1, Figure S1: ^1^H NMR spectra of (a) COG and (b) MACOG, Figure S2: ATR-FTIR spectra of castor oil (CO), glycerol (gly), COG, and MACOG, Table S1: ATR-FTIR peak assignments for castor oil (CO), glycerol, COG, and MACOG.
