# Supplementary material for: Biodegradable Polymeric Foams Based on Modified Castor Oil, Styrene, and Isobornyl Methacrylate
Source: Polymers (Basel). 2021 Jun 4;13(11):1872. doi: 10.3390/polym13111872 (PMC8200243; doi:10.3390/polym13111872)
Supplement: Supplementary file 1 [file polymers-13-01872-s001.zip › polymers-1246896-supplementary.pdf]

Supplementary Materials

Biodegradable Polymeric Foams Based on Modified Castor Oil, Styrene, and Isobornyl Methacrylate

James Dicks * and Chris Woolard

Methods

Synthesis of castor oil glycerides

60.0 g of castor oil and 13.1 g of glycerol (1:2.2 mole ratio) were charged in a three necked 500 ml round bottom flask equipped with inert gas inlet (Ar), thermometer, Liebig condenser and mechanical stirrer. The mixture was heated to 180 °C. 0.5 phr $Ca(OH)_2$ was added to the mixture. The mixture was heated to 200 °C for 1.5h. After 1.5h the reaction vessel was plunged into salted ice water to rapidly cool to 15 °C whilst stirring rapidly. Any excess glycerol remained in the mixture and no further purification was performed. The product was a golden-brown liquid at room temperature.

Synthesis of maleated castor oil gycerides

To the castor oil glycerides (COG) previously prepared, 59.0 g of MA was added in the reaction vessel. Hydroquinone (0.10 phr) and N,N-DMBA (0.15 phr) were added as free radical inhibitor and accelerant, respectively. The mixture was heated in an oil bath to 100 °C with continuous stirring under inert atmosphere (Ar) for 3.5 h. MA that sublimed on the reaction vessel walls was regularly melted back into the reaction mixture by pouring hot silane oil over the outside of the vessel. At the end of the reaction, any excess MA remained in the mixture, and the product was used for polymerization without any further purification. The product was a yellow/brown highly viscous liquid at room temperature.

Results and Discussion

Synthesis of maleated castor oil glycerides

The synthesis of MACOG was performed by a two-step "one-pot" procedure. Firstly, COG was produced via a base catalyzed glycerolysis with castor oil and glycerol, using $Ca(OH)_2$ as catalyst. The COG product had an acid value (AV) of 8.54 ± 0.0048 mg KOH/g, and a saponification value (SV) of 143.27 ± 0.82 mg KOH/g, resulting in 0.0025 ± 1.46×10^{-5} moles of ester groups per gram of COG. The maleation reaction was performed for 3.5 h, at which point the product had an AV of 258.16 ± 0.74 mg KOH/g, and SV of 552.66 ± 2.66 mg KOH/g, resulting in 0.0053 ± 4.74×10^{-5} moles of ester bonds per gram of MACOG.

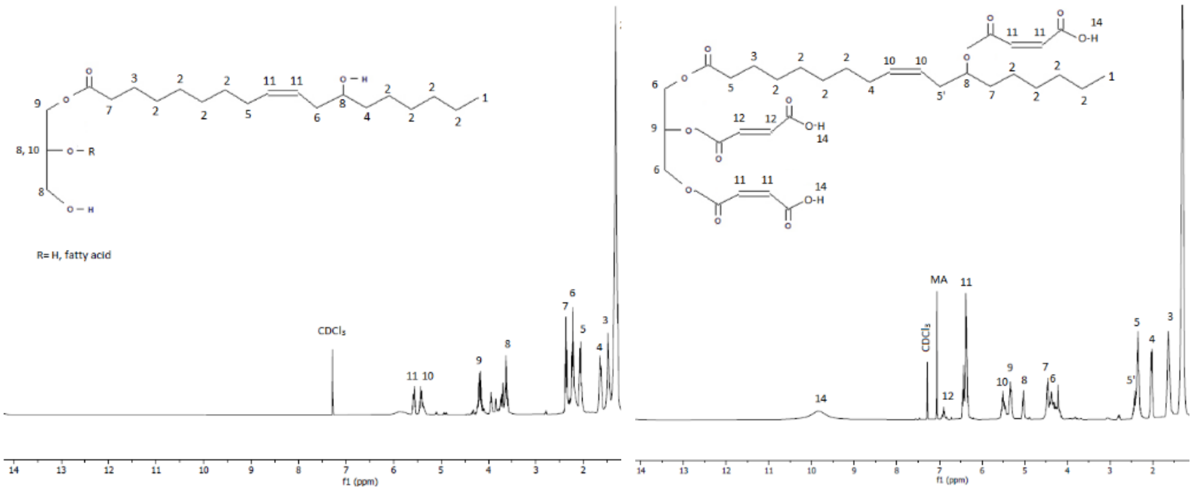

(a) **(b)**

Figure S1: ^{1}H NMR spectra of a) COG and b) MACOG.

For COG, ^{1}H NMR was performed using $CDCl_3$ solvent at a frequency of 400.22 MHz at 293.4K over 8 scans, presented in Figure S1a. A triplet at 0.89 ppm related to terminal –CH3, a multiplet in the range of 1.25-1.40 ppm related to –CH2- on the fatty acid, a quartet at 1.47 ppm related to -CH2-CH(OH), a triplet at 1.65 ppm related to β-CH2-CH2-(CO)O-, a quartet at 2.07 ppm and a triplet at 2.23 ppm related to CH2-CH2-CH=CH- and -CH=CH-CH2-CH(OH), respectively, and a multiplet in the range of 2.26-2.41 related to α–CH2(CO)O-. Due to COG being a mixture of predominantly monoglyceride and diglyceride with a minor fraction of triglyceride and glycerol, there were various hydroxyl groups and esters present on the glycerol centre. A multiplet in the range of 3.54-3.76 ppm related to CH–OH and CH2-OH on the glycerol centre and CH(OH) on the fatty acid.[1] A quartet of triplets at 4.18 ppm related to CH2-O(CO)- methylene on the glycerol centre, a multiplet at 5.34-5.47 ppm related to –CH-O(CO)- methine attached to the glycerol centre and a multiplet in the range of 5.52-5.63 ppm related to –CH=CH- unsaturation on the fatty acid.[2-5]

For MACOG, ^{1}H NMR was performed using $CDCl_3$ solvent at a frequency of 400.22 MHz at 293.3 K over 8 scans, presented in Figure S1b. A triplet at 0.85-0.93 ppm related to terminal –CH3, a quintet at 1.30 ppm related to –CH2- on the fatty acid, a triplet at 1.63 ppm related to β–CH2-CH2(CO)O-, a quartet at 2.02 ppm related to CH2–CH2-CH=CH-. A multiplet in the range of 2.36-2.49 ppm related to α–CH2(CO)O- and –CH=CH-CH2-CHO(CO)-, a multiplet in the range of 4.24-4.42 ppm related to CH2-O(CO)- methylene on the glycerol centre, a quartet at 4.46 ppm related to CH2-CH2-CH-O(CO) on the fatty acid, a quartet at 5.03 ppm related to CH-O(CO)- maleate on the fatty acid, a quartet at 5.32 ppm related to –CH=CH- unsaturation on the fatty acid, and a triplet of doublets at 5.5 ppm related to –CH-O(CO)- methine attached to the glycerol centre. A multiplet in the range of 6.30-6.49 ppm related to –CH=CH- of the maleate group and a multiplet in the range of 6.83-6.96 ppm related to –CH=CH- of the fumarate group. The singlet at 7.06 ppm related to unreacted MA. The broad singlet at 9.83 ppm related to the –COOH- on the maleate/fumarate groups.[3, 6] Comparative to COG, the peaks related to CH(OH), CH-OH and CH2-OH diminished for MACOG, which suggested that the majority of the hydroxyl groups present on COG were consumed during the maleation reaction. Furthermore, new peaks appeared for MACOG related to CH-O(CO)- and CH2-O(CO)- of maleates/fumarates appear, suggesting that the esterification reaction was successful.[4, 7, 8]

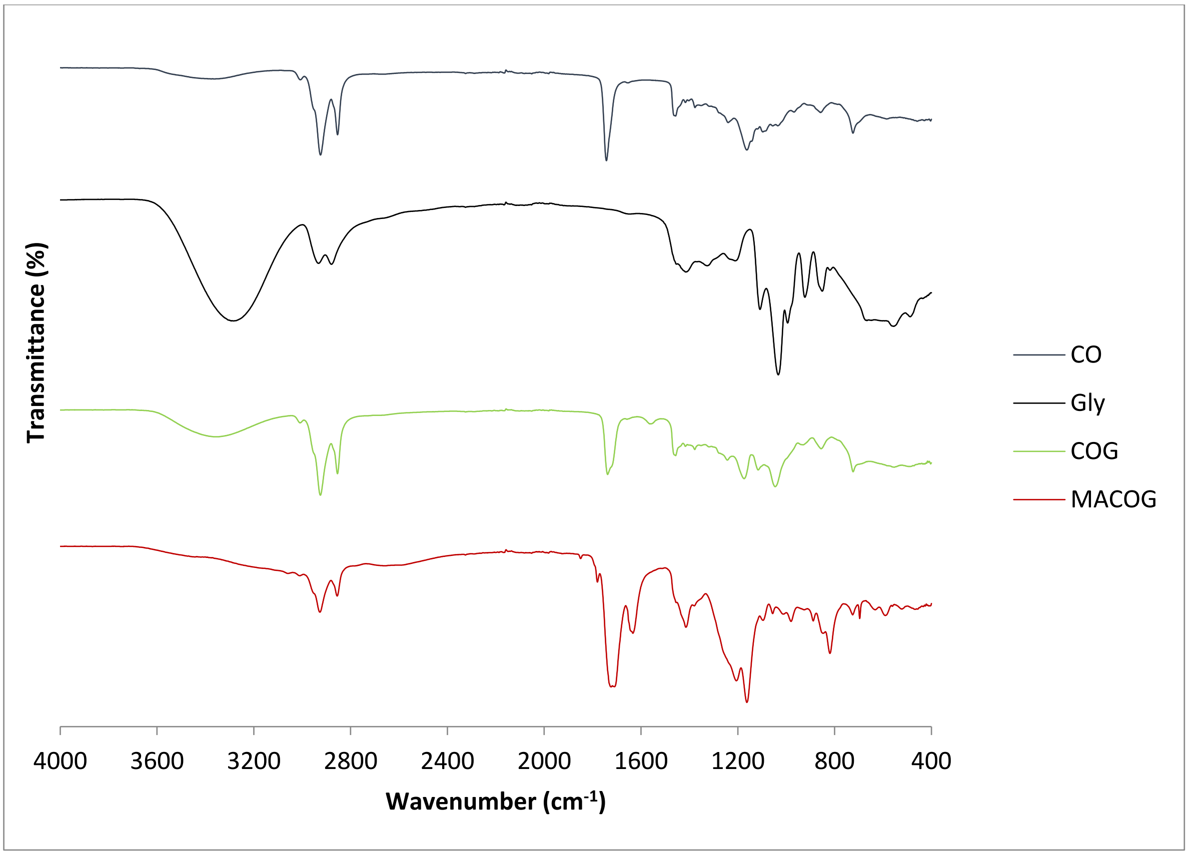

Figure S2: ATR-FTIR spectra of castor oil (CO), glycerol (gly), COG, and MACOG.

Table S1. ATR-FTIR peak assignments for castor oil (CO), glycerol, COG, and MACOG.

	CO	Glycerol	COG	MACOG	Ref.
ν(O-H)	3364	3283	3362	-	[9-11]
ν_{as}(C-H2)	2924	2932	2924	2926	[11-13]
ν_{s}(C-H2)	2853	2878	2854	2856	[11-13]
ν(C=O)	1742	-	1737	1723	[10, 14, 15]
ν(-C=C-)	1633	-	1633	1633	[16]
ν(C-O-H)	1240	1226		-	[2, 14, 15, 17]
ν(C-O) and δ(C-H)	1162	-	1165	1159	[2, 14, 15]
ν(C-O-H) secondary	1095	1108	1115	-	[18, 19]
ν(C-O-H) primary	-	1032	1045	-	[18, 19]
ϒ(O-H)	858	851 + 650-600	855 + 650-600	-	[17, 20]
ϱ(C-H2)	723	-	723	725	[15]

The ATR-FTIR spectra of castor oil (CO), glycerol, COG, and MACOG are presented in Figure S2, with some of the common absorption peak assignments summarized in Table S1. Additional absorption peaks were also present for the aforementioned compounds. For glycerol, the peak at 1326 cm^{-1} was assigned to the δ(C-H) of the methine [20]. For COG, the intensity of the hydroxyl ν(O-H) at 3362 cm^{-1} increased compared to castor oil, due to the relative increase in hydroxyl group functionality on the product. The peak at 1563 cm^{-1} was assigned to the ν(COO^-) of the carboxylate salt, which was a soap product formed during the glycerolysis reaction with $Ca(OH)_2$ and free fatty acids [9, 21-24]. An additional peak related to ν(C-O) of primary hydroxyl groups was observed at 1045 cm^{-1}, which was not present on the castor oil triglyceride spectrum.

For MACOG, the peak due to ν(O-H) in the 3500 cm^{-1} region present on COG diminished, and was replaced by ν(O-H) for maleate acid groups with a broad peak between 3400-2500 cm^{-1} [25]. This was indicative that the majority of hydroxyl groups were consumed during the anhydride esterification

reaction and the formation of maleate half esters occurred [6]. Weak peaks at 1853 cm^{-1} and 1783 cm^{-1} were indicative that there was a cyclic anhydride present, although the weak absorption intensity suggested that the majority of MA was successfully consumed [8, 26, 27]. The peak at 1723 cm^{-1} was assigned to the carbonyl valence bond ν(C=O), which was more intense than that of castor oil and COG and indicated more ester groups were present due to the formation of maleate half-esters. Moreover, it shifted down from 1742 cm^{-1} for castor oil due to the influence of the carbonyl contribution of the carboxylic acid groups on the maleate half esters. Relative to castor oil and COG, an increase in intensity of the peak at 1633 cm^{-1} assigned to the ν(C=C) indicated the presence of double bonds from the maleate groups on the product [27]. The peak at 1414 cm^{-1} was assigned to the carboxyl ϱ(C-O-H) [28]. The peak at 1052 cm^{-1} was assigned to the O-C-C ester group vibration [18]. This peak could also be due to the CO-O-CO vibration of the residual anhydride, although these are typically seen as broad peaks [29]. It has previously been suggested that the C=C-H out of plane bending vibration for the *trans* alkene fumarate group has a peak around 980 cm^{-1} [18, 30]. The presence of a peak in this area further implied that maleate groups may have isomerized to fumarates. The peak at 890 cm^{-1} was assigned to ω($C-H_2$). The peak at 819 cm^{-1} was attributed to the ϒ(C=C-H) of the maleate group [5, 6]. The weak peak at 696 cm^{-1} was assigned to ϒ(C=C-H) in the *cis* conformation related to unsaturation on the fatty acid moieties [29].

References

[1] J. Lu, P. Wang, Q. Wang, Y. Wang, M. Jiang, Validated ^{1}H and (13)C nuclear magnetic resonance methods for the quantitative determination of glycerol in drug injections, Molecules 23(5) (2018) 1177.

[2] L. Zhang, M. Zhang, L. Hu, Y. Zhou, Synthesis of rigid polyurethane foams with castor oil-based flame retardant polyols, Industrial Crops and Products 52 (2014) 380-388.

[3] A. Campanella, J.J. La Scala, R.P. Wool, The use of acrylated fatty acid methyl esters as styrene replacements in triglyceride‐based thermosetting polymers, Journal of Polymer Engineering 49(12) (2009) 2384-2392.

[4] D.A. Echeverri, W.A. Perez, L.A. Rios, Synthesis of maleated-castor oil glycerides from biodiesel-derived crude glycerol, Industrial Crops and Products 49 (2013) 299-303.

[5] D.A. Echeverri, L.A. Rios, B.L. Rivas, Synthesis and copolymerization of thermosetting resins obtained from vegetable oils and biodiesel-derived crude glycerol, European Polymer Journal 67 (2015) 428-438.

[6] E. Can, R. Wool, S. Küsefoğlu, Soybean and castor oil based monomers: Synthesis and copolymerization with styrene, Journal of Applied Polymer Science 102(3) (2006) 2433-2447.

[7] M.A. Saied, S.H. Mansour, A.A. Ward, I.S. Rahim, H.A. Zayed, A.L. Saad, K.N. Abdel Nour, Characterization of maleated vegetable oils for insulation purposes and agricultural applications, Polimery 59(10) (2014).

[8] H.J. Wang, M.Z. Rong, M.Q. Zhang, J. Hu, H.W. Chen, T. Czigány, Biodegradable foam plastics based on castor oil, Biomacromolecules 9(2) (2007) 615-623.

[9] S. Pathan, S. Ahmad, Synthesis, characterization and the effect of the s-triazine ring on physico-mechanical and electrochemical corrosion resistance performance of waterborne castor oil alkyd, Journal of Materials Chemistry A 1(45) (2013) 14227-14238.

[10] M. Zhang, H. Pan, L. Zhang, L. Hu, Y. Zhou, Study of the mechanical, thermal properties and flame retardancy of rigid polyurethane foams prepared from modified castor-oil-based polyols, Industrial Crops and Products 59 (2014) 135-143.

[11] R. Gawad, V. Fellner, Evaluation of glycerol encapsulated with alginate and alginate-chitosan polymers in gut environment and its resistance to rumen microbial degradation, Asian-Australasian journal of animal sciences 32(1) (2019) 72.

[12] M. Kachel-Jakubowska, A. Matwijczuk, M. Gagoś, Analysis of the physicochemical properties of post-manufacturing waste derived from production of methyl esters from rapeseed oil, International Agrophysics 31(2) (2017) 175.

[13] N.N. Mahamuni, Y.G. Adewuyi, Fourier transform infrared spectroscopy (FTIR) method to monitor soy biodiesel and soybean oil in transesterification reactions, petrodiesel− biodiesel blends, and blend adulteration with soy oil, Energy & Fuels 23(7) (2009) 3773-3782.
[14] R. Müller, G. Wilke, Research, Synthesis and radiation curing of acrylated castor oil glycerides, Journal of Coatings Technology 11(6) (2014) 873-882.
[15] H. Li, S. Niu, C. Lu, Pyrolysis characteristics of castor oil through thermogravimetric coupled with Fourier transform infrared spectroscopy, Procedia Engineering 205 (2017) 3705-3710.
[16] P. Samyn, D. Van Nieuwkerke, G. Schoukens, L. Vonck, D. Stanssens, H. Van Den Abbeele, Quality and Statistical Classification of Brazilian Vegetable Oils Using Mid-Infrared and Raman Spectroscopy, Applied Spectroscopy 66(5) (2012) 552-565.
[17] B. Sharma, Instrumental Methods of Chemical Analysis, Krishna Prakashan Media, Subhash Nagar, India, 1981.
[18] M. Mitha, M. Jayabalan, Studies on biodegradable and crosslinkable poly (castor oil fumarate)/poly (propylene fumarate) composite adhesive as a potential injectable biomaterial, Journal of Materials Science: Materials in Medicine 20(1) (2009) 203.
[19] W.G. de Ruig, Infrared Spectra of Monoacid Triglycerides; With Some Applications to Fat Analysis, Pudoc, Centre for Agricultural Publishing and Documentation, Wageningen, 1971.
[20] J. Coates, Interpretation of infrared spectra, a practical approach, Encyclopedia of Analytical Chemistry: Applications, Theory and Instrumentation (2006).
[21] S. Pathan, S. Ahmad, s-Triazine ring-modified waterborne alkyd: Synthesis, characterization, antibacterial, and electrochemical corrosion studies, ACS Sustainable Chemistry & Engineering 1(10) (2013) 1246-1257.
[22] C. Liu, J. Li, W. Lei, Y. Zhou, Development of biobased unsaturated polyester resin containing highly functionalized castor oil, Industrial Crops and Products 52 (2014) 329-337.
[23] S. Zheng, Y. Chen, M.A. Brook, Thermoplastic silicone elastomers based on Gemini ionic crosslinks, Polymer Chemistry (2020).
[24] S. Kongjao, S. Damronglerd, M. Hunsom, Purification of crude glycerol derived from waste used-oil methyl ester plant, Korean Journal of Chemical Engineering 27(3) (2010) 944-949.
[25] J. Cavusoglu, G. Çaylı, Polymerization reactions of epoxidized soybean oil and maleate esters of oil ‐ soluble resoles, Journal of Applied Polymer Science 132(7) (2015).
[26] X. Mamat, Y. Wang, W. Eli, New potentially environmentally friendly copolymer of styrene and maleic acid—castor oil monoester, Polymers for Advanced Technologies 23(9) (2012) 1271-1275.
[27] R.S. Alves, D.L. Maia, F.A. Fernandes, F.X. Feitosa, H.B. de Sant'Ana, Synthesis and application of castor oil maleate and castor oil maleate-styrene copolymers as demulsifier for water-in-oil emulsions, Fuel 269 (2020) 117429.
[28] M. Mosiewicki, M.I. Aranguren, J. Borrajo, Mechanical properties of linseed oil monoglyceride maleate/styrene copolymers, Journal of Applied Polymer Science 97(3) (2005) 825-836.
[29] D.L. Pavia, G.M. Lampman, G.S. Kriz, J.A. Vyvyan, Introduction to Spectroscopy, 5 ed., Cengage Learning, Boston, Massachusetts, 2008.
[30] B.L. Grunden, C.S.P. Sung, Cure characterization of unsaturated polyester resin by near-IR and mid-IR spectroscopy, Macromolecules 36(9) (2003) 3166-3173.
